# Alzheimer’s Disease Pharmacotherapy in Relation to Cholinergic System Involvement

**DOI:** 10.3390/biom10010040

**Published:** 2019-12-26

**Authors:** Gabriela Dumitrita Stanciu, Andrei Luca, Razvan Nicolae Rusu, Veronica Bild, Sorin Ioan Beschea Chiriac, Carmen Solcan, Walther Bild, Daniela Carmen Ababei

**Affiliations:** 1 Grigore T. Popa” University of Medicine and Pharmacy, Center for Advanced Research and Development in Experimental Medicine (CEMEX), 16 Universitatii street, 700115 Iasi, Romania; gabriela-dumitrita.s@umfiasi.ro (G.D.S.); andrei.g.luca@umfiasi.ro (A.L.); 2 Grigore T. Popa” University of Medicine and Pharmacy, Pneumology Department, 16 Universitatii Street, 700115 Iasi, Romania; 3 Grigore T. Popa” University of Medicine and Pharmacy, Pharmacodynamics and Clinical Pharmacy Department, 16 Universitatii street, 700115 Iasi, Romania; razvan.nicolae.rusu@gmail.com (R.N.R.); dana.ababei@gmail.com (D.C.A.); 4 University of Agricultural Sciences and Veterinary Medicine “Ion Ionescu de la Brad”, Faculty of Veterinary Medicine, 8 M. Sadoveanu Alley, 700489 Iasi, Romania; carmensolcan@yahoo.com; 5 Grigore T. Popa” University of Medicine and Pharmacy, Department of Physiology, 16 Universitatii street, 700115 Iasi, Romania; waltherbild@gmail.com

**Keywords:** Alzheimer’s disease, cholinergic system, cholinesterase inhibitors, acetylcholinesterase, butyrylcholinesterase

## Abstract

Alzheimer’s disease, a major and increasing global health challenge, is an irreversible, progressive form of dementia, associated with an ongoing decline of brain functioning. The etiology of this disease is not completely understood, and no safe and effective anti-Alzheimer’s disease drug to prevent, stop, or reverse its evolution is currently available. Current pharmacotherapy concentrated on drugs that aimed to improve the cerebral acetylcholine levels by facilitating cholinergic neurotransmission through inhibiting cholinesterase. These compounds, recognized as cholinesterase inhibitors, offer a viable target across key sign domains of Alzheimer’s disease, but have a modest influence on improving the progression of this condition. In this paper, we sought to highlight the current understanding of the cholinergic system involvement in Alzheimer’s disease progression in relation to the recent status of the available cholinesterase inhibitors as effective therapeutics.

## 1. Introduction

Alzheimer’s disease (AD), known as the leading form of dementia in the aging people, is described by a global cognitive decline that involves memory loss, visuospatial orientation, impaired judgment, communication, and reasoning; it is a major and growing health challenge worldwide [1,2]. According to the World Health Organization (WHO), globally, over 30 million people are afflicted by AD and this number is projected to double every two decades to reach 66 million in 2030 and about 115 million by 2050. The total estimated worldwide cost of caring for AD patients in 2018 was estimated to be $1 trillion, corresponding to a global gross domestic product of 1.2%, and this figure will rise to $ 2 trillion annually by 2030; costs that may weaken social and economic development and could overwhelm health and social services [3]. This multifactorial disease is no longer a problem just for high developed countries. More than two-thirds of all dementia patients live in India, Brazil, and China, and that percentage will increase to three quarters by 2050. The high individual, familial, social, and financial burden of AD requires bold and critical plans. As a result, most states and many international organizations have elaborated strategies and analytical reports to solve the problem [4]; and numerous studies based on mathematical models or on qualified estimates are trying to predict the development of AD in people [5,6,7,8]. A recent research regarding predictions of AD treatment and care costs in European countries using computer simulations, and the evolution of the costs of treating and caring of AD patients associated with the introduction of drug deliveries at different stages of the disease revealed that any involvement extending the length of a patient’s ‘stay’ in the mild, moderate, or severe stage of the disease will lead to added costs of care by 2080, increasing the proportion of people with AD. Thus, prolonging lifespan is essential in terms of refining the quality of life of patients, and the introduction of novel compounds should consider the supplementary costs imposed upon society [9].

To date, the central neuropathological hallmarks noticed in AD are extracellular amyloid beta (Aβ) plaques, a consequence of aberrant transmembrane amyloid precursor protein (APP) cleavage, accumulation of large intracellular quantities of neurofibrillary tangles, cerebral amyloid angiopathy, glial cell dysfunction, synaptic, and neuronal injury [10,11]. In recent years, a large body of research has described a series of different pathogenic mechanisms underlying the causes of AD comprising cholinergic neuronal degeneration, the amyloid cascade hypothesis, τ protein phosphorylation, neuroinflammation, oxidative stress, calcium theory, dysregulation of iron metabolism, reduced glucose utilization, imperfect insulin signaling, abnormal cholesterol homeostasis, and mitochondrial dysfunction [12,13,14,15,16,17].

According to the cholinergic theory, the development of Alzheimer’s disease symptoms is mainly related to structural alterations in cholinergic synapses, loss of specific subtypes of acetylcholine (ACh) receptors, the death of ACh-generating neurons, and, consequently, the deterioration of cholinergic neurotransmission. These issues lead to a relative accumulation of the ACh-hydrolyzing enzyme, acetylcholinesterase (AChE) [18]. 

There are no effective therapeutic options for the vast majority of patients, and principal causes of the disease are still unidentified, except for a minor number of familial cases produced by genetic mutations. Pharmacotherapeutic strategies for the AD treatment are mainly classified in the following ways: (i) Therapies that inhibit the onset of this condition by isolating the principal progenitors; (ii) disease-modifying treatments termination or the reversal of disease progression; and (iii) symptomatic treatments that act on the cognitive signs of the disease, protecting patients from further cognitive failure [2,18].

The current pharmacotherapeutic approach based on cholinesterase inhibitors offer a feasible therapeutic target for partial stabilization of cognitive function, improvement of quality of life, and moderation the burden care in AD patients, as cholinergic neuron damage represents a constant and primary finding in disease states [1,19]. However, these compounds have positive results for a short period of time, usually 1–3 years, and they cannot influence the disease evolution [20]. The paper provides a brief summary of the current understanding of the cholinergic system involvement in Alzheimer’s disease progression in relation to the recent status of the available cholinesterase inhibitors, as effective therapeutic options.

## 2. The Cholinergic Hypothesis of Alzheimer’s Disease

Support for the cholinergic hypothesis in Alzheimer’s disease came in the mid-1970s with studies highlighting significant neocortical deficits of the choline acetyltransferase (ChAT), the enzyme responsible for the Ach synthesis [21]. Subsequent findings of decreased choline uptake, ACh release and lack of cholinergic perikarya from the basal frontal lobe confirmed an important presynaptic cholinergic impairment [22]. Cholinergic neurotransmission has been shown to be implicitly involved in a number of major psychic processes such as memory, learning, waking and sleep, stress response, and affectivity [14,23]. 

Regarding the alteration of the cholinergic system of brain neurotransmitters, there has been research on the activity of the enzyme ChAT and on the ratio between the choline uptake and the ChAT level, which is not significantly increased except in some brain regions, but which is generally lower than the brain in control subjects, as well as on the possibility of optimizing cerebral acetylcholine level by using acetylcholine precursors or cholinesterase inhibitors. At the cellular level, the neuronal choline deficiency initially suggested by Wurtman [24] and attested by some facts that a 40%–50% decrease in cerebral acetylcholine level, a decrease in choline-dependent membrane constituents, and increase in its metabolites, is considered a pathogenic element for dementia and AD. It has been hypothesized that, under conditions of choline deficiency, for the recovery of the necessary choline, a true "auto-cannibalism" of the membranous choline, which favors cell membrane to break with the release of enzymes that cause an abnormal proteolysis of the β-amyloid precursor protein and the formation of amyloid protein β-A4, a highly harmful complex by the effect of neuronal degeneration, that this amyloid has on a primary culture of neurons [14,22,24,25]. The pathogenic hypothesis regarding choline deficiency has at least two corollaries: One diagnostic, by the possibility of determining the concentration of choline derivatives in cerebrospinal fluid (CSF), and the other therapeutic by using potassium channel inhibitors in order to enhance the synthesis of choline and increase acetylcholine uptake. The efficacy of therapeutic growth of cerebral acetylcholine level is possible due to the relative preservation of muscarinic acetylcholine receptors, especially M1, even in the areas affected by hypoperfusion and hypometabolism [26,27].

The most important neurochemical deficiency in AD is acetylcholine impairment. The anatomical basis of cholinergic deficits is atrophy and degeneration of subcortical cholinergic neurons, especially those in the basal frontal lobe (nucleus basalis magnocellularis of Meynert) that provide cholinergic innervation of the cerebral cortex. The selective deficiency of acetylcholine in AD, as well as the observation that central cholinergic antagonists such as atropine can induce a confusing state that results in similarities of Alzheimer’s with dementia, have led to the emergence of the cholinergic hypothesis that acetyl deficiency critical in the onset of the symptoms of AD [14]. Acetylcholine was the first neurotransmitter identified and used by all cholinergic neurons with an important role in the central and peripheral nervous system. Both pre- and post-ganglionic parasympathetic neurons, pre-ganglionic sympathetic neurons, and some post-ganglionic sympathetic neurons use acetylcholine as a neurotransmitter. Cholinergic synapses are widespread in the central nervous system. They probably behave just like those in the periphery. With a major distribution in the brain, cholinergic neurotransmission is responsible for modulating neuronal functions [21,23].

### 2.1. Acetylcholine—The Neurotransmitter of The Cholinergic Synapse: Synthesis, Depolarization, Release, Inactivation, and Recovery

**Figure 1:**
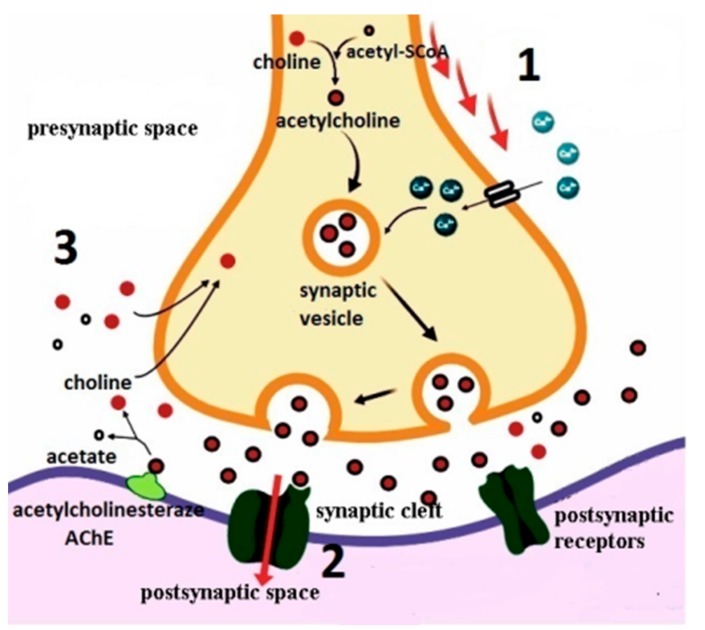
Schematic representation of the acetylcholine release course and cholinergic hypothesis of AD. (1) Action potential causing influx of Ca^2+^ and subsequent membrane docking of synaptic vesicles; (2) acetylcholine binds to receptors initiating a graded depolarization in the post synaptic cell; (3) AChE catalyzes the breakdown of acetylcholine and choline molecules are reabsorbed by the presynaptic neuron. ACh, acetylcholine; AChE, acetylcholinesterase; Acetyl-CoA, acetyl coenzyme A.

Cholinergic neurotransmission is based on proteins involved in acetylcholine synthesis, storage, transport, and degradation. This is synthesized in the cytoplasm of cholinergic neurons from choline and active acetate at about 20% and most (80%) synthesizing at the terminal buttons [28]. Choline originates from outside the neuron (results from lipid degradation, especially from lecithin, as well as from acetylcholine hydrolysis) and is captured by axonal termination through the intervention of a specific transport mechanism. Acetyl-coenzyme A (acetyl-CoA) is formed from pyruvate in the mitochondria. Choline esterification with the acetyl radical is catalyzed by ChAT, a soluble enzyme, present in high concentration in the cytoplasm of cholinergic nerve endings, and its activity is regulated by neuronal depolarization, influx of calcium ions, and phosphorylation of the enzyme (Figure 1) [22,23,28].

The release of acetylcholine is done by exocytosis of synaptic vesicles. They fuse with the pre-synaptic membrane, eliminating its neurotransmitter content in the synaptic cleft where it can activate both muscarinic and nicotinic receptors. The membranes of the embedded vesicles temporarily enlarge the surface of the neuronal membrane, and then undergo an endocytosis process, restoring the vesicle formation in the cytoplasm of nerve cell termination [14,29]. The nerve impulse greatly increases the release of the neurotransmitter acetylcholine, a biologically active amount. The release activity is due to the influx of calcium ions, which occurs as a result of the opening of the slow channels in the pre-synaptic membrane, controlled by depolarization [14,22]. Acetylcholine crosses the synaptic cleft, protected in part by the enzyme that hydrolyzes it, AchE, either due to the existence of some channels in the transmitter, or to the inhibition by the excess of the substrate or by certain components of the post-synaptic membrane. The acetylcholine molecule comprises a cationic end, which enters an anionic site of the active receptor surface and an ester group, which is fixed by an esterophilic site of this surface [30]. Acetylcholine that breaks down from the cholinergic receptor complex is rapidly hydrolyzed and inactivated under the influence of acetylcholinesterase (true cholinesterase), an enzyme found in the synaptic cleft, probably linked to the basal lamina. Following the hydrolysis of acetylcholine to choline, its collection takes place in presynaptic cholinergic neurons through an active transport system [23,31].

AchE has been shown to be the most important therapeutic target for the symptomatic improvement of AD, since its inhibition was considered to be achievable as a therapeutic target. There are two types of cholinesterase, acetylcholinesterase (mainly present in blood and neuronal synapses) and butyrylcholinesterase (mainly in the liver) and the difference between the two is represented by substrates [19,25]. Acetylcholinesterase exists in two general classes of molecular forms: Simple homomeric oligomers of catalytic subunits (monomers, dimers, and tetramers) and heteromeric associations of catalytic subunits with structural subunits. Homomeric forms are present in the cell as soluble species, probably intended for export or for association with the other cell membrane, usually by an attached glycophospholipide group [32]. A heteromeric form largely present in neuronal synapses is a tetramer of disulfide-linked catalytic subunits of a 20,000-Da subunit bound to a lipid and located on the outer surface of the cell membrane. The other heteromeric form consists of tetramers of catalytic subunits, linked by disulfide bridges to each of the three chains of subunits with a collagen-like structure. This molecular species with a molecular mass of about 106 Da is associated with the basal lamina of the skeletal muscle base plate. Molecular cloning has shown that a single gene encodes vertebrate acetylcholinesterase’s [19,21,22,31]. However, the products of multiple genes arise from alternative mRNA processing that differs only by its carboxylic terminus. The portion of the gene encoding the catalytic center of the enzyme is invariable. Therefore, it is expected that the different types of acetylcholinesterase will have identical inhibitory substrate and specificities [28,32]. 

Cholinesterase’s are a family of proteins that share a structure of α, β-hydrolases. The family includes some esterase’s, other hydrolases, which are not present in the nervous system, and surprisingly, proteins without hydrolase activity such as thyroglobulin and members of the tact and neuroligin protein families [33]. The tyrosine is further away from the active center, but probably contributes to the stabilization of some ligands. The catalytic triad, the cholinergic subzone, and the acyl pocket are located at the base of the depression, while the peripheral area is at the edge of the depression. The depression has a depth of 18–20Å with centrosymmetric basis relative to subunits.

The catalytic mechanism is similar to that of other hydrolases; the hydroxyl group of the serine becomes highly nucleophilic by a charge-retransmission mechanism involving the carboxylate anion of glutamate, the imidazole anion of histidine, and the hydroxyl anion of the serine. During the enzymatic attack on acetylcholine, which is an ester with trigonal geometry, a tetrahedral intermediate is formed between the enzyme and the substrate [14,30]. Acetylcholine decomposes to an acetyl conjugate of the enzyme with concomitant release of choline. Acetyl CoA is highly labile to hydrolysis after which acetate and the active enzyme are formed. AchE is one of the most efficient enzymes known: One molecule of AchE can hydrolyze 6x105 molecules of acetylcholine/minute; thus, the recovery time of the enzyme is 100 microseconds [25,29]. 

Cholinergic neurons in the central nervous system (CNS) were localized using ChAT antibodies. Cholinergic neurons have been described: (i) At the level of the basal nucleus, with projection in the cerebral cortex; (ii) in the septum, with projection in the hippocampus and neocortex; (iii) in the pedunculo-pontine and lateral tegmental nuclei, with thalamus projection; (iv) unstressed (small local neurons).

### 2.2. Cholinergic Receptors 

Cholinergic receptors are "active" proteins of the membranous structures that delimit the synaptic space being able to couple with acetylcholine, triggering a series of specific transformations at the membrane or cytoplasmic level. Acetylcholine, a neurotransmitter with modulator functions in the CNS, acts by activating the receptors causing stimulation or inhibition, depending on the neuronal type and localization of the receptor. The acetylcholine receptors (cholinergic receptors) have been designated according to the selective pharmacological ligands (muscarinic and nicotine), in muscarinic and nicotinic. The cellular effectors of these receptors are sodium ion channels and membrane proteins modulated by G proteins [14,19,25].

*Muscarinic receptors* are part of a complex system that includes G protein and secondary messengers. They differ markedly from nicotinic ones both functionally and structurally; they are glycoproteins with molecular weights around 80 kDa, located at the membrane level as serpentines, being formed of three portions, coupled with G protein, capable of modulating a wide variety of ion channels [34]. Based on pharmacological and genetic cloning methods, five subtypes of muscarinic receptors were identified: M1, M2, M3, M4, and M5. M1 receptors are located postsynaptic in the neurons of the CNS (of the cortex, hippocampus, striatum, and basal nuclei), of the vegetative lymph nodes and of the stomach. The active internal pole of the M1 receptor is coupled to the G-stimulatory protein (Gq), which belongs to the G family [35]. These receptors act synergistically with the M3 receptors, producing a series of effects that can affect cognitive processes. M2 receptors are located on the postsynaptic membrane (myocardium and brain) and the presynaptic membrane (acting as auto-receptors) and at the active pole these receptors are coupled with an inhibitory G protein (Gi) [14]. M3 receptors present at central level, especially in cerebellar structures, and their active end is coupled with Gq protein. M4 receptors generally located presynaptic, act as auto-receptors, and regulate the release of Ach or as heteroreceptors that modulate synaptic transmission. The M5 receptors are situated predominantly at the peripheral level (smooth musculature) [29,33]. The stimulation of muscarinic receptors by the physiological agonist Ach, or by the pharmacological ones of muscarinic type, influences two effector systems and two metabolic pathways: Adenylatcyclase and phospholipase C. Phospholipase C (PLC) is modulated by Gq protein, followed by activation of PLC that catalyzes metabolism [25,34]. Phosphatidylinositol diphosphate with the formation of secondary messengers: Inositoltrophosphate (IP3) and diacylglycerol (DAG). IP3 releases calcium deposited in the cytoplasmic reticulum, calcium reaching the cytoplasm forms a complex with calmodulin, and activates calcium-calmodulin kinase with a role in phosphorylation of intracytoplasmic proteins; a process of cellular stimulation takes place. Adenylatcyclase modulated by protein (Gi) causes its inhibition, thus decreasing the biosynthesis and concentration of cyclic adenosine monophosphate (cAMP), intracellular secondary messenger, as well as inhibition of calcium channels with diminished cellular excitability [18,29,35].

*Nicotinic receptors* are located at different structures, including the CNS, lymph nodes, and muscles. Each receptor is composed of five different subunits: α, β, γ (fetal), δ, and ε (adult) and functions as an ion channel with a ligand-regulated gate [36]. Three types of nicotinic receptors have been identified: N1, N2, and N3. Those located in muscles and lymph nodes (N1 and N2) are composed of two subunits α and one β, γ, and δ, and those located at the CNS (N3) level are formed by combining two types of subunits [37]. There are various complex forms of nicotinic receptors, but in the central nervous system, only two subtypes are more representative: One consisting of 4α2β subunits with high affinity for nicotine and cytosine and another subtype of five 7α subunits with lower affinity for nicotine, with subunit 7α being the most widespread [36,37]. Each nicotinic receptor may have different properties and functions. In the CNS, most of the nicotinic receptors are expressed at the level of the presynaptic neuronal membrane and have the role of regulating the release of neurotransmitters (including acetylcholine), following the stimulation process with the increased presynaptic calcium concentration [14]. N3 receptors realize not only the retrieval and transmission of information from one neuron to another, but also its processing. Numerous studies have shown that the levels of these receptors change with age, so their number is higher in the early stages of embryonic development, which suggests that these receptors may play an important role in growth, development, and aging [29]. At the CNS level, they are distributed in a high percentage in the prefrontal cerebral cortex, the hippocampus, the basal nucleus, and the cross-linked thalamic nucleus. The dysfunction of these receptors was associated with Alzheimer’s dementia, receptors that regulate neuronal plasticity, differentiation, proliferation, apoptosis, and clearance of older neurons [19]. Also, subtype 7α has a high permeability for calcium ions with increasing its level and activating kinase protein that regulates gene expression and protein level leading to neuronal structural and functional changes. The presynaptic activation of these receptors is followed by membrane depolarization, with increased exocytosis and massive release of neurotransmitters (acetylcholine, dopamine, serotonin, glutamate, gamma-aminobutyric acid) [38,39].

## 3. Cholinesterase Inhibitors, A Positive Approach to Delay the Progress of Alzheimer’s Disease

**Figure 2:**
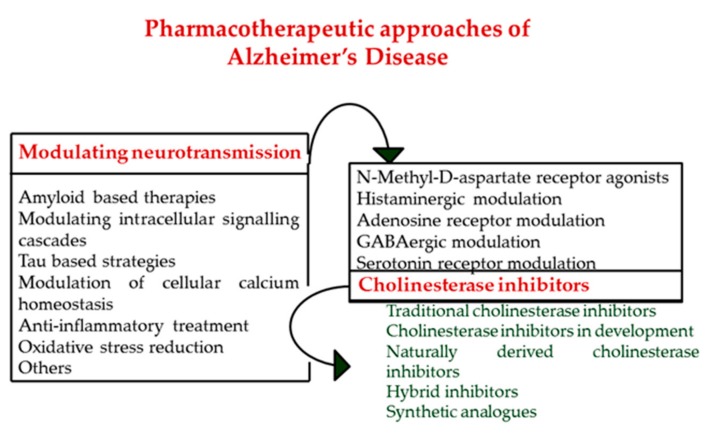
Schematic diagram of pharmacological approaches of Alzheimer’s disease therapies in relation to the main pathogenic mechanisms including cholinergic neuronal degeneration, the amyloid cascade hypothesis, imperfect insulin signalling, τ protein phosphorylation, calcium theory, neuroinflammation, oxidative stress, or others (dysregulation of iron metabolism, reduced glucose utilization, abnormal cholesterol homeostasis, and mitochondrial dysfunction).

Recognized as a multifactorial condition that involves multiple biological mechanisms, the etiology of Alzheimer’s disease is not completely understood, and no safe and effective compound to prevent, stop, or reverse its evolution is currently available. Figure 2 schematically shows some therapeutic strategies that over time have been investigated for the treatment of AD. Existing AD therapies focused on agents that aimed to increase the cerebral acetylcholine levels by facilitating cholinergic neurotransmission through inhibiting cholinesterase. These compounds, known as cholinesterase inhibitors, offer a viable target across key sign domains of Alzheimer’s disease [40,41,42,43].

**Figure 3:**
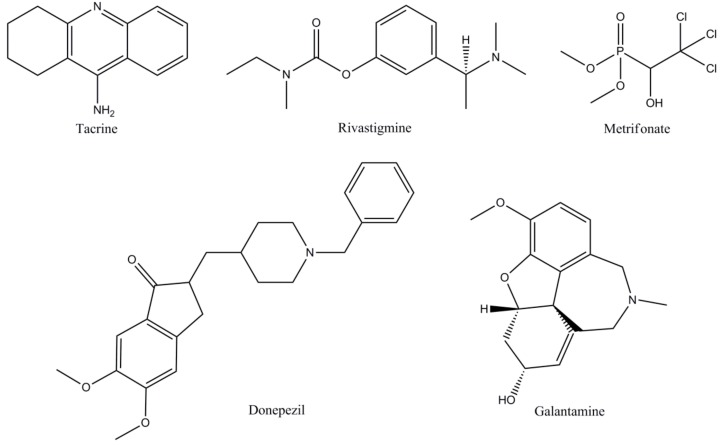
Traditional cholinesterase inhibitors (ChEIs). The molecular structures of compounds approved for treatment of Alzheimer’s disease.

**Figure 4:**
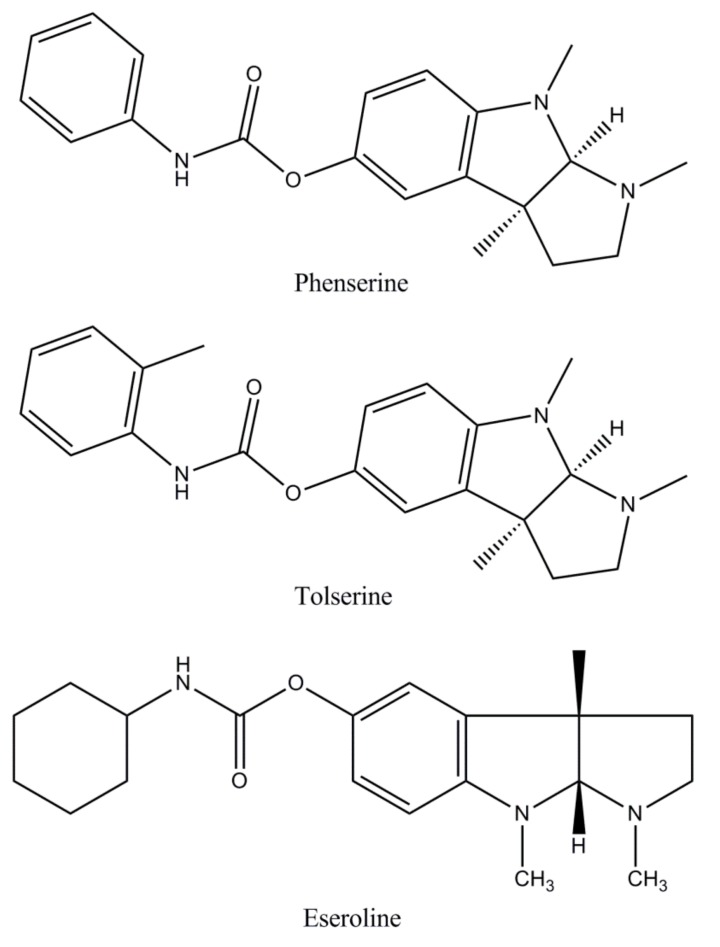
The structures of ChEIs in development: Phenserine, Tolserine, and Eseroline.

**Figure 5:**
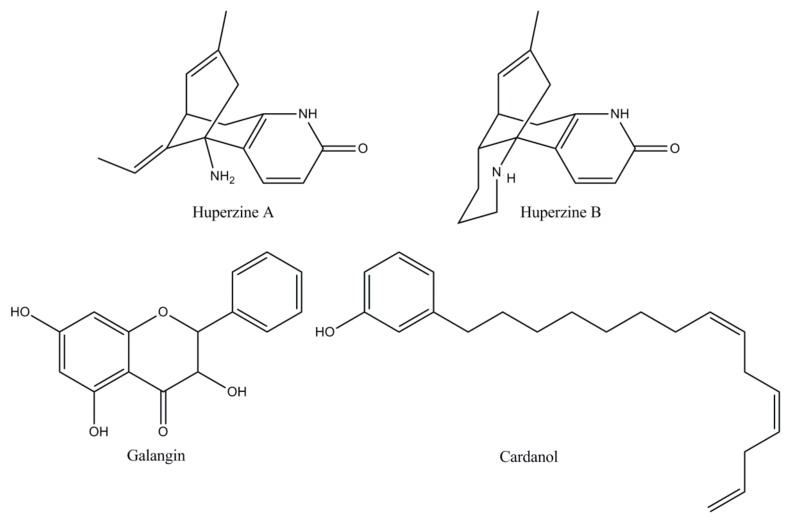
The molecular structures of naturally derived ChEIs: Huperzine A and B, Galangin, and Cardanol.

**Figure 6:**
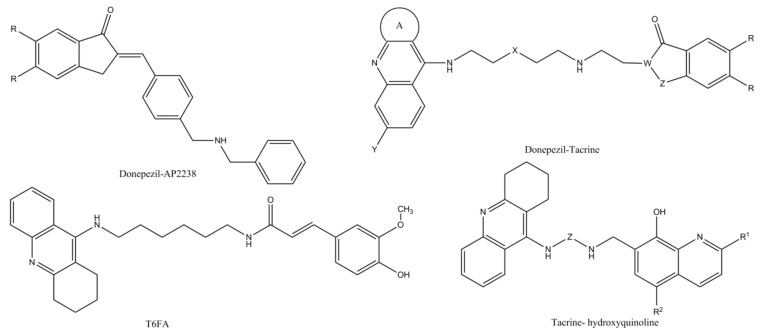
Hybrid ChEIs. The molecular structures of donepezil–tacrine hybrid, tacrine–ferulic acid (T6FA) hybrid, and tacrine and 8-hydroxyquinoline hybrids.

**Figure 7:**
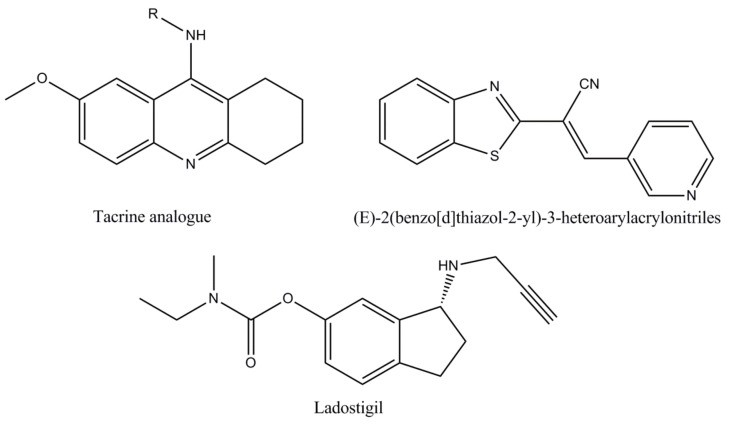
The molecular structures of synthetic analogues: Tacrine analogues, (*E*)-2(benzo[d]thiazol-2-yl)-3-heteroarylacrylonitriles and ladostigil.

The most important types of cholinesterase’s in the mammalian brains are represented by acetylcholinesterase (AChE) and butyrylcholinesterase (BuChE) differing in genetics, structure, and kinetics. AChE is predominantly observed in the neuronal synapses and blood; whereas at the level of the human brain, BuChE is located close to glial cells and neurons or in tangles and neuritic plaques in Alzheimer’s individuals [2,19]. While in patients with AD, AChE activity is progressively reduced, BuChE activity increases slightly. Most of the cholinesterase inhibitors that are presented for AD therapy target both AChE and BuChE [44]. In Table 1, the pharmacological characteristics of the main cholinesterase inhibitors (ChEIs) are presented: Traditional ChEIs (Figure 3), ChEIs in development (Figure 4), naturally derived ChEIs (Figure 5), hybrid ChEIs (Figure 6), and synthetic analogues (Figure 7). 

In AD therapy, ChEIs are dosed in two stages, an initial dose-escalation phase to obtain a therapeutic dose and a maintenance phase in which the therapeutic dose is administered for long-term therapy [80]. ChEIs may discreetly delay the loss of brain function in mild to moderate AD patients. However, these compounds may also determine a variety of side effects as a consequence of cholinergic stimulation in diverse zones of the brain and the periphery. Centrally mediated acute gastrointestinal events (especially nausea and vomiting) are class side effects of all ChEIs with the dual AChE and BuChE action and are described mainly throughout the dose-escalation phase of the treatment. Instead, these effects can be minimized by using slow dose escalation with low dose gradations and administering them with food [81]. The side effects of ChEIs registered frequently during the maintenance phase of therapy include central nervous system signs; extrapyramidal events; sleep disorders and cardiorespiratory disturbance related to cholinergic action in the cortex, caudate nucleus of the basal ganglia, brainstem, and medulla; cardiorespiratory symptoms and urinary incontinence in relation to peripheral cholinergic action [19,25]. ChEIs are well tolerated; patient’s compliance and caregiver suitability are good when dosed with care. The promising tolerability and safety profiles of these compounds make them appropriate first-line therapy strategies for AD [18,81]. 

The blood–brain barrier (BBB) represents an important obstacle in the efficient administration of therapeutic compounds at the cerebral level, imposing size and biochemical limitations on the passage of molecules. Over time, a large number of strategies have been explored to overcome the BBB, instead quite limited by lack of specificity, safety issues and failure to reach adequate concentrations of released agents to appropriate volumes of brain tissue [82,83]. Moreover, according to the vascular hypothesis of AD, deterioration of the blood vessels represent the primary insult, causing blood–brain barrier (BBB) dysfunction and reduced cerebral perfusion that, in turn, lead to neuronal damage and Aβ accumulation in the brain [84,85,86]. In this context, commonly used drugs may be retrieved differently by the AD patient’s brain compared to the non-AD patients. Changes in AD regarding this aspect are determined by the properties of the BBB, CSF reabsorption, brain blood flow, and P-glycoprotein 1 (P-gp) permeability, known as the multidrug-resistance protein 1-P-gp, also known as multi-drug resistance-associated protein 1-P-gp; these changes are the most studied, and some of these effects will continue to increase while others decrease the balance of medication inside the brain. These differences could lead to changes in the efficacy, potency, therapeutic window, side effects on CNS, and doses of commonly used drugs [87,88]. In the case of diminished CSF reabsorption, drugs may remain in the CNS longer. Decreased blood flow in the brain will only affect those drugs that enter the CNS rapidly. Administration of cholinesterase inhibitors such as donepezil is probably dependent on brain flow and a decrease in it will result in reduced absorption and thus reduced therapeutic effect [89]. Other in vivo pharmacokinetic studies have highlighted the capacity of donepezil and its fragments to cross the BBB by passive diffusion, as previously confirmed by other researchers who reported that the concentrations of donepezil in the brain expressed as the area-under-the-curve of the concentration, exceed plasma concentrations in mice, rats, and rabbits [90,91]. A series of studies showed that high doses of donepezil (23 mg) were more effective than lower doses (5 and 10 mg) and that pharmacological effects depended not only on dose increase and drug formulation, but also on the mechanism of active efflux along the blood–brain barrier, P-glycoprotein being a determining factor in the efflux of donepezil from the central compartment to the bloodstream [92,93].

The tacrine compound is rapidly taken up in the brain, being located in the cortex, hippocampus, thalamus, and striated bodies, but its distribution does not correlate with the distribution of AChE, with tacrine’s 10-fold higher concentration at the brain level compared to plasma, tacrine being a non-selective, reversible inhibitor of central and peripheral AChE [94,95]. From 1998 to 2018, 100 drugs have been tested in clinical trials and only four have been approved for clinical use. Due to the numerous failures and the large number of evidences attesting to the complex pathologies of the disease, the researchers resorted to a multi-target-based approach based on combining different drugs into a single formulation, obtaining a hybrid molecule that simultaneously exerts its individual effects. In this regard, hybrid anti-cholinesterase molecules were created in the form of multi-target ligands against AD. In this respect, hybrids were obtained, such as tacrine hybrids, whose selectivity over cholinesterase inhibition is markedly superior to tacrine, agents whose molecules have also demonstrated the ability to cross the BBB [96,97]. The BBB acts as a dynamic interface between the CNS and peripheral tissues, this barrier becoming a therapeutic target for providing drugs needed for neuropsychiatric disorders such as Alzheimer’s dementia. Therefore, regulating BBB function may be a new therapeutic target for treating AD.

## 4. Different Preclinical Models to Study Cholinergic Hypothesis of Alzheimer’s Disease 

There are different models used in animal research to reproduce Alzheimer’s disease. The classic ones are administration of intracerebral β-amyloid injections or transgenic mice overexpressing β-amyloid or presenilin1 [98]. Moreover, aging animals or induction of brain inflammation can lead to production of AD animal models. Separately, glucose and energy metabolism impairment, phosphatase inhibition, or lesions of the forebrain cholinergic system complete the models used in preclinical studies. The models that relay on cholinergic system impairment were based on clinical observations that connected memory deficit with the cholinergic neurons, nuclei or pathways which were later, by development of techniques to visualize these structures, associated directly with the forebrain nuclei. Damage at this level is done by administration of 192 IgG-saporin, which leads to a destruction of cholinergic neurons and thus lead to the animal model of AD [99]. Currently, each animal model of AD has its own limitations, which researchers have to take into account. For example, this specific model is not associated with the production of neurofibrillary tangles just A13 deposition in rabbit models instead of A8 plaques. Also, it does not reproduce a laniary decrease in cognitive deficit as it should, but rather just a stationary one. Even though these limitations are present, certain breakthroughs such as the development of cholinesterase inhibitors, some of the most effective treatment in AD symptoms, could not have been developed [98,99,100].

Lately, research brings new observation regarding the physiopathology of this disease, such as degeneration of choline acetyltransferase neurons in the vertical diagonal band of Broca, a change that occurs in the early stage, and is responsible for the innervation of newly generated immature neurons in mice models [101]. Moreover, it has been demonstrated in differentiated rat pheochromocytoma PC12 cells against amyloid-beta induced cytotoxicity, that nerve growth factor plays an important role in the loss of cholinergic neurons [102]. These observations lead to new treatment hypothesis such as theta burst stimulation, direct delivery of nerve growth factor to the brain using porous silicon carriers, or deep magnetic stimulation applied to mice models of 5XFAD mice in order to enhance neuron regeneration [103]. In a similar manner, by administering venous blood from young mice to aged AD mice, it has been shown that this has neuroprotective effects and therapeutic effects over the hippocampal cholinergic input [104]. In addition to these findings, intervention during the perinatal period in APP/PS1 mice by choline supplementation demonstrated a beneficial effect over AD progression during adult age [62,105].

Natural compounds, such as β-Amyrin, were tested for ameliorating memory deficit and decreasing neurogenesis impairments in Aβ-injected AD mice models [106]. Similarly, *Dendropanax morbifera* was tested in Aβ peptide-treated mice in order to produce brain neuron damage and on cognitive deficit in neuronal cell, showing benefic effects [107] the same way *Salicornia europaea L.* administration demonstrated anti-neuroinflammatory and ameliorative potential in scopolamine-induced amnesic C57/BL6N mice [108]. These observations are also sustained by years of clinical expertise and observations. Epidemiological studies, for example, showed that, in some patients, although they had AD changes in the brain, they preserved cognitive functions, and thus, in combination with hippocampal sections from mice, researchers demonstrated that the cholinergic neural activation from the medial septal nucleus is a main modulating system and that cholinergic agonists with effect over M1 muscarinic receptor can prevent suppression of neuronal activity [109,110,111,112]. In regards to these findings, a novel selective allosteric M1 muscarinic and sigma-1 receptor agonist named AF710B tested on 13-month-old Tg rats showed promising results in AD management [113]. Currently, more in-depth research is being conducted in different mice strains regarding gene alterations that occur in AD, such as the changes present in the TgCRND8 mice, which are related to cholinergic neuron dysfunction and have found differences between this strain and the wild type in regards to actively translating mRNAs in anterior forebrain cholinergic [114], which could help us develop new strains closer in resemblance to that of AD patients [113]. Other models such as APPPS1 mice [115] or 3xTg-AD are being explored or are currently used in laboratory testing [116].

Taking into consideration all those described above, animal models are of paramount importance in understanding the mechanisms of AD, and since new findings are constantly emerging together with the developments in clinical medicine, new animal models and measurements regarding AD must continuously be developed, improved, and studied more in depth.

## 5. From Single Targets towards Multi-Target Directed Ligands

The concept “one molecule-one target-one disease” fails to provide a comprehensive solution for AD treatment due to the complex and multilayered nature of the disease [117]. As a result, nowadays the anti-AD drug research is moving towards a new approach that addresses the various pathways involved in the onset and evolution of this pathology—multi-target-directed ligands (MTDLs) [118]. The novel molecules come with additional challenges such as a fine binding affinity of ligands for multiple targets, favorable pharmacological profile, neuroprotective properties, and drug-like activities, thus suggesting a good BBB permeability [119]. Apart from cholinesterase inhibition, which remains the current effective therapeutic option for symptomatic relief, some other activities can be inserted into the MTDLs to design and produce an extensive variety of possible dual- and multi-acting anti-AD compounds [119,120,121]. 

The cholinesterase inhibitors that interact simultaneously with AChE (catalytic and peripheral sites) and Aβ plaque deposition coupled with added properties such as antioxidant action [122], neuroprotective or voltage-dependent calcium channel antagonistic activity [123,124,125,126], histamine H3 receptor antagonism [127,128,129,130], cannabinoid CB1 receptor antagonism [131,132], and beta-site APP cleaving enzyme (BACE1) inhibition [70,125,133,134] display the potential of ameliorating the cognitive deficit in AD by restoring cholinergic activities [135,136,137].

An important group of novel compounds based on a series of benzothiazolylureas scaffolds targeting amyloid-binding alcohol dehydrogenase (ABAD) have revealed an inhibitory action at cellular level with controlled cytotoxicity, exhibiting a kinetic mechanical action of reversible mixed inhibition. These results, with further optimization, could lead to a new class of therapeutic agents for AD [138,139].

## 6. Concluding Remarks

A large body of studies highlight that Alzheimer’s disease is a brain disorder, multifactorial in nature, involving various biological mechanisms; it is still an important and growing health challenge worldwide. In the last decades, the cholinergic hypothesis has dominated the pathogenesis and pathophysiology, following a series of research focused on improving the cerebral levels of acetylcholine by inhibiting cholinesterase, thus facilitating cholinergic neurotransmission. The cholinergic approaches offer the most viable therapeutic target across fundamental sign domains of Alzheimer’s disease, but have a modest influence on improving the progression of this condition. Future pharmacotherapies should not be limited to the postulates of the cholinergic hypothesis. Novel AD strategies should be centered on developing or repurposing drugs with the ability to target multiple disease features such as risk factors, mechanism-based versus non-mechanism-based approaches, symptomatic therapies, and lifestyle changes.

## Figures and Tables

**Table 1:** Pharmacological characteristics of the principal cholinesterase inhibitors.

Drug		Pharmacological Aspects
**Traditional ChEIs**	*Tacrine* [25,45,46,47]	The first compound approved in 1993 for the Alzheimer’s patient’s therapy, tacrine is a non-competitive, rapidly reversible inhibitor of both AChE and BuChE. The bioavailability of tacrine varieties from 17-37%, elimination half-life ranges from 1.3 to 7.0 hours and is almost 75% protein bound. Metabolism of tacrine is achieved by CYP 450 1A2 and 2D6.
	*Donepezil* [20,25,48,49]	Approved in 1996 for mild to moderate AD therapy, donepezil is a non-competitive, rapidly reversible AChE inhibitor. Bioavailability of 100%, readily absorbed after oral administration, vastly protein bound - 96%, with an excretion half-life of 60–90 hours. Donepezil is mainly metabolized by CYP 450 isoenzymes 2D6 and 3A4.
	*Rivastigmine* [49,50,51,52]	Approved in 2000 for AD treatment, the compound is considered a non-competitive, pseudo-irreversible of both AChE and BuChE with equal proficiency. Bioavailability of rivastigmine is quite low at 40%, protein binding at 40%, and elimination half-life of almost 2 hours. Rivastigmine is rapidly and primarily metabolized by cholinesterase’s.
	*Galantamine* [19,53,54,55]	A competitive, rapidly reversible potent AChE inhibitor, galantamine was approved in AD therapy in 2001. It is well absorbed with an 85% to 100% bioavailability in oral delivery. Plasma protein binding of this compound is about 18%, with a half-life of 7 hours. The main way for galantamine metabolism occurs through CYP isoenzymes 2D6 and 3A4.
	*Metrifonate* [19,56,57]	Metrifonate a long-acting irreversible inhibitor of ChEI is not an approved AD drug because of risk of neuromuscular transmission dysfunction and respiratory paralysis. Even if metrifonate has proven a strong and important clinical effect it was abandoned after Phase III.
**ChEIs in development**	*Phenserine* [58,59,60]	Phenserine is a pure non-competitive, selective AChE inhibitor, being a promising agent for developing new strategies in AD therapy. It has a half-life of 10 minutes and owns an action more than 8 hours, being highly penetrative to the brain with a brain: Plasma concentration ratio of 10:1.
	*Tolserine* [19,25,61]	A partial non-competitive, reversible AChE inhibitor, tolserine, has a pharmacokinetics half-life of 12 minutes and a pharmacodynamics half-life greater than 8 hours. Tolserine delivers a selectivity for AChE of 200-fold versus 75-fold for phenserine. The 29-methyl substitution additionally raises the hydrophobic properties of tolserine compared to phenserine improving its blood– brain barrier permeability, ensuring high brain absorption (brain:blood ratio 24:1).
	*Eseroline* [25,62,63]	Considered un metabolite of physostigmine, eseroline has a competitive, limited, and reversible effect on AChE inhibition. Zhan et al. [63] found that a cyclic alkyl carbamate derived from eseroline is more effective against AChE with a great selectivity compared with BChE. There have been no recent studies reporting the effect of eseroline.
**Naturally derived ChEIs**	*Huperzine* [18,64]	A Lycopodium alkaloid extracted from the Chinese medicinal plant *Huperzia serrata*, huperzine is an effective, reversible, and very selective AChE inhibitor. Huperzine has an oral bioavailability of more than 96% metabolized by CYP1A2, with a possible secondary influence by CYP3A1/2. In China it is the drug selected for AD therapy. In the USA, Phase II studies have shown a modest but clinically substantial effect on the cognition of AD patients.
	*Flavonoid* [19,25,65]	Flavonoids have attracted attention due to their free radical scavenging properties highlighting the ability to influence cognition and learning in humans and AD animal models. Galangin, a flavonol extracted and isolated from the rhizomes of *Alpiniae officinarum*, confirmed the most important inhibitory activity against AChE. Nevertheless, the toxicity of these alternative candidates for Alzheimer’s therapy has not been explored in preclinical studies and no clinical trials have been described to date.
	*Cardanol* [66,67,68]	Cardanol derivatives as new potential candidates of AChE inhibitors designed from nonisoprenoid phenolic lipids of cashew (NIPLs) of *Anacardium occidentale* nut-shell liquid have revealed favorable results. The development of cardanol derivatives seems to be attractive because of the abundance of the source of raw materials. However, there are no reported preclinical and toxicity studies, to date.
**Hybrid ChEIs**	*Donepezil–AP2238 hybrid* [69,70,71]	The first inhibitor capable of binding to both the catalytic and peripheral sites of AChE, AP 2238 hybrid has an activity against AChE similar to that of donepezil but with a higher capacity to inhibit Aβ-mediated toxicity. At present, there are no reports of human studies, not being preclinical and clinical safety and toxicity.
	*Donepezil–tacrine hybrid* [72,73]	Designed and synthetized from a unit of 6-chlorotacrine and 5,6-dimethoxy- 2-[(4-piperidinyl)methyl]-1-idanone moiety of donepezil, donepezil–tacrine hybrids have proven to be highly potent inhibitors of both AChE and BChE as well as beta-amyloid aggregation determined by AChE.
	*Tacrine–ferulic acid (T6FA) hybrid* [74]	In vitro studies have shown that T6FA can significantly inhibit auto- and AChE aggregation of Aβ (1-40), blocking Aβ-induced cell death (1-40) in PC12 cells. Moreover, in an AD rodent model, T6FA considerably enriched the cognitive capacity along with growing ChAT and superoxide dismutase activity, reducing AChE activity.
	*Tacrine and 8-hydroxyquinoline hybrids*	Designed and synthesized by Fernandez-Bachiller et al. [75], the new hybrids have been shown to be more effective than tacrine against ChEIs inhibition. The compounds revealed low cell level toxicity, antioxidant, and copper-complexing activities.
**Synthetic analogues**	*Tacrine analogues* [76,77,78]	Screening results revealed that most tacrine analogues displayed important inhibition against AChE compared to tacrine.
	*(E)-2(benzo[d]thiazol-2-yl)-3-heteroarylacrylonitriles* [25,76]	Developed as an inhibitor of AChE since 2012, the compound (E)-2(benzo[d]thiazol-2-yl)-3-heteroarylacrylonitriles has been shown to be more selective for AChE than galanthamine.
	*Ladostigil* [19,25,79]	Derived from the combination of two pharmacophores: The carbamate moiety of rivastigmine and the propargyl group of rasagiline, ladostigil represents a novel anti-AD compound, which combines neuroprotective proprieties with brain selective monoamine oxidase and cholinesterase inhibitory activities. The drug is currently included in a Phase IIb clinical trial for the AD therapy and comorbid dementia associated with extrapyramidal conditions and depression.

## Abbreviations

AD	Alzheimer’s disease
WHO	World Health Organization
AChE	acetylcholinesterase
AChEIs	acetylcholinesterase inhibitors
BuChE	butyrylcholinesterase
ChEIs	cholinesterase inhibitors
CYP	cytochrome system
USA	United States of America
Aβ	amyloid beta
APP	amyloid precursor protein
ACh	acetylcholine
CSF	cerebrospinal fluid
ChAT	choline acetyltransferase
acetyl-CoA	acetyl-coenzyme A
CNS	central nervous system
Gq	G-stimulatory protein
PLC	Phospholipase C
IP3	inositoltrophosphate
DAG	diacylglycerol
cAMP	cyclic adenosine monophosphate
BBB	blood brain barrier
P-gp	P-glycoprotein 1
MTDLs	multi-target-directed ligands
BACE 1	β-secretase, β-site APP cleaving enzyme 1
ABAD	amyloid-binding alcohol dehydrogenase

## References

[1] Ghumatkar P.J., Patil S.P., Jain P.D., Tambe R.M., Sathaye S. (2015). Nootropic, neuroprotective and neurotrophic effects of phloretin in scopolamine induced amnesia in mice. Pharmacol. Biochem. Behav..

[2] Deture M.A., Dickson D.W. (2019). The neuropathological diagnosis of Alzheimer’s disease. Mol. Neurodegener..

[3] Alzheimer’s Disease International (2018). World Alzheimer Report 2018—The State of the Art of Dementia Research: New Frontiers.

[4] Maresova P., Mohelska H., Dolejs J., Kuca K. (2015). Socio-economic Aspects of Alzheimer’s Disease. Curr. Alzheimer Res..

[5] Brookmeyer R., Johnson E., Ziegler-Graham K., Arrighi H.M. (2007). Forecasting the global burden of Alzheimer’s disease. Alzheimer Dement..

[6] Ferri C.P., Prince M., Brayne C., Brodaty H., Fratiglioni L., Ganguli M., Hall K., Hasegawa K., Hendrie H., Huang Y. (2005). Global prevalence of dementia: A Delphi consensus study. Lancet.

[7] Prince M., Bryce R., Albanese E., Wimo A., Ribeiro W., Ferri C.P. (2013). The global prevalence of dementia: A systematic review and metaanalysis. Alzheimer Dement..

[8] Gustavsson A., Green C., Jones R.W., Förstl H., Simsek D., de Reydet de Vulpillieres F., Luthman S., Adlard N., Bhattacharyya S., Wimo A. (2017). Current issues and future research priorities for health economic modelling across the full continuum of Alzheimer’s disease. Alzheimer Dement..

[9] Cimler R., Maresova P., Kuhnova J., Kuca K. (2019). Predictions of Alzheimer’s disease treatment and care costs in European countries. PLoS ONE.

[10] Serrano-Pozo A., Frosch M.P., Masliah E., Hyman B.T. (2011). Neuropathological alterations in Alzheimer disease. Cold Spring Harb. Perspect. Med..

[11] Singh S.K., Srivastav S., Yadav A.K., Srikrishna S., Perry G. (2016). Overview of Alzheimer’s disease and some therapeutic approaches targeting A β by using several synthetic and herbal compounds. Oxid. Med. Cell. Longev..

[12] Swerdlow R.H. (2018). Mitochondria and Mitochondrial Cascades in Alzheimer’s Disease. J. Alzheimer Dis..

[13] Perez S.E., Ginsberg S.D., Counts S.E., Mufson E.J. (2009). Cholinergic system during the progression of Alzheimer’s disease: Therapeutic implications. Expert Rev. Neurother..

[14] Ferreira-Vieira T., Guimaraes I., Silva F., Ribeiro F. (2016). Alzheimer’s disease: Targeting the Cholinergic System. Curr. Neuropharmacol..

[15] Oshiro S., Morioka M.S., Kikuchi M. (2011). Dysregulation of Iron Metabolism in Alzheimer’s Disease, Parkinson’s Disease, and Amyotrophic Lateral Sclerosis. Adv. Pharmacol. Sci..

[16] Gibson G., Cotman C., Lynch G., Blass J. (2017). Calcium Hypothesis of Alzheimer’s disease and brain aging: A framework for integrating new evidence into a comprehensive theory of pathogenesis. Alzheimer Dement..

[17] Gamba P., Staurenghi E., Testa G., Giannelli S., Sottero B., Leonarduzzi G. (2019). A crosstalk between brain cholesterol oxidation and glucose metabolism in Alzheimer’s disease. Front. Neurosci..

[18] Martinez A., Castro A. (2006). Novel cholinesterase inhibitors as future effective drugs for the treatment of Alzheimer’s disease. Expert Opin. Investig. Drugs.

[19] Mehta M., Adem A., Sabbagh M. (2012). New Acetylcholinesterase Inhibitors for Alzheimer’s Disease. Int. J. Alzheimer Dis..

[20] Sun Y., Lai M., Lu C., Chen R. (2008). How long can patients with mild or moderate Alzheimer’s dementia maintain both the cognition and the therapy of cholinesterase inhibitors: A national population-based study. Eur. J. Neurol..

[21] Nestor P.J., Scheltens P., Hodges J.R. (2004). Advances in the early detection of Alzheimer’s disease. Nat. Rev. Neurosci..

[22] Francis P.T., Palmer A.M., Snape M., Wilcock G.K. (1999). The cholinergic hypothesis of Alzheimer’s disease: A review of progress. J. Neurol. Neurosurg. Psychiatr..

[23] Chen X.Q., Mobley W.C. (2019). Exploring the pathogenesis of Alzheimer disease in basal forebrain cholinergic neurons:Converging insights from alternative hypotheses. Front. Neurosci..

[24] Wurtman R.J. (1994). American Society for Clinical Investigation. J. Clin. Investig..

[25] Sharma K. (2019). Cholinesterase inhibitors as Alzheimer’s therapeutics (Review). Mol. Med. Rep..

[26] Doody R.S., Stevens J.C., Beck C., Dubinsky R.M., Kaye J.A., Gwyther L., Mohs R.C., Thal L.J., Whitehouse P.J., DeKosky S.T. (2001). Practice parameter: Management of dementia (an evidence-based review): Report of the quality standards subcommittee of the American Academy of Neurology. Neurology.

[27] Richter N., Nellessen N., Dronse J., Dillen K., Jacobs H.I.L., Langen K.J., Dietlein M., Kracht L., Neumaier B., Fink G.R. (2019). Spatial distributions of cholinergic impairment and neuronal hypometabolism differ in MCI due to AD. NeuroImage Clin..

[28] Picciotto M.R., Higley M.J., Mineur Y.S. (2012). Acetylcholine as a Neuromodulator: Cholinergic Signaling Shapes Nervous System Function and Behavior. Neuron.

[29] Talesa V.N. (2001). Acetylcholinesterase in Alzheimer’s disease. Mech. Ageing Dev..

[30] Rees T.M., Brimijoin S. (2003). The role of acetylcholinesterase in the pathogenesis of Alzheimer’s disease. Drugs Today.

[31] Francis P.T. (2005). The Interplay of Neurotransmitters in Alzheimer’s Disease. CNS Spectr..

[32] Tabet N. (2006). Acetylcholinesterase inhibitors for Alzheimer’s disease: Anti-inflammatories in acetylcholine clothing!. Age Ageing.

[33] Sanabria-Castro A., Alvarado-Echeverría I., Monge-Bonilla C. (2017). Molecular Pathogenesis of Alzheimer’s Disease: An Update. Ann. Neurosci..

[34] Jiang S., Li Y., Zhang C., Zhao Y., Bu G., Xu H., Zhang Y.W. (2014). M1 muscarinic acetylcholine receptor in Alzheimer’s disease. Neurosci. Bull..

[35] Pavía J., De Ceballos M., Sanchez De La Cuesta F. (1998). Alzheimer’s disease: Relationship between muscarinic cholinergic receptors, β-amyloid and tau proteins. Fundam. Clin. Pharmacol..

[36] Lombardo S., Maskos U. (2015). Role of the nicotinic acetylcholine receptor in Alzheimer’s disease pathology and treatment. Neuropharmacology.

[37] Oddo S., LaFerla F.M. (2006). The role of nicotinic acetylcholine receptors in Alzheimer’s disease. J. Physiol. Paris.

[38] Buckingham S.D., Jones A.K., Brown L.A., Sattelle D.B. (2009). Nicotinic acetylcholine receptor signalling: Roles in alzheimer’s disease and amyloid neuroprotection. Pharmacol. Rev..

[39] Georgi S. (2005). Nicotinic Acetylcholine Receptors and Alzheimer’s Disease Therapeutics: A Review of Current Literature. J. Young Investig..

[40] Cao J., Hou J., Ping J., Cai D. (2018). Advances in developing novel therapeutic strategies for Alzheimer’s disease 11 Medical and Health Sciences 1109 Neurosciences. Mol. Neurodegener..

[41] Folch J., Petrov D., Ettcheto M., Abad S., Sánchez-López E., García M.L., Olloquequi J., Beas-Zarate C., Auladell C., Camins A. (2016). Current Research Therapeutic Strategies for Alzheimer’s Disease Treatment. Neural Plast..

[42] Graham W.V., Bonito-Oliva A., Sakmar T.P. (2017). Update on Alzheimer’s Disease Therapy and Prevention Strategies. Annu. Rev. Med..

[43] Frozza R.L., Lourenco M.V., de Felice F.G. (2018). Challenges for Alzheimer’s disease therapy: Insights from novel mechanisms beyond memory defects. Front. Neurosci..

[44] Giacobini E. (2003). Cholinesterases: New Roles in Brain Function and in Alzheimer’s Disease. Neurochem. Res..

[45] Harel M., Schalkt I., Ehret-Sabatiert L., Bouett F., Goeldnert M., Hirtht C., Axelsen P.H., Silmanii I., Sussman J.L. (1993). Quaternary ligand binding to aromatic residues in the active-site gorge of acetylcholinesterase. Proc. Natl. Acad. Sci. USA.

[46] Farlow M., Gracon S.I., Hershey L.A., Lewis K.W., Sadowsky C.H., Dolan Ureno J. (1992). A Controlled Trial of Tacrine in Alzheimer’s Disease. JAMA J. Am. Med. Assoc..

[47] Watkins P.B., Zimmerman H.J., Knapp M.J., Gracon S.I., Lewis K.W. (1994). Hepatotoxic Effects of Tacrine Administration in Patients With Alzheimer’s Disease. JAMA J. Am. Med. Assoc..

[48] Grossberg G.T. (2003). Cholinesterase Inhibitors for the Treatment of Alzheimer’s Disease: Getting On and Staying On. Curr. Ther. Res..

[49] Jann M.W., Shirley K.L., Small G.W. (2002). Clinical pharmacokinetics and pharmacodynamics of cholinesterase inhibitors. Clin. Pharmacokinet..

[50] Inglis F. (2002). The tolerability and safety of cholinesterase inhibitors in the treatment of dementia. Int. J. Clin. Pract. Suppl..

[51] Onor M.L., Trevisiol M., Aguglia E. (2007). Rivastigmine in the treatment of Alzheimer’s disease: An update. Clin. Interv. Aging.

[52] Bentham P., Gray R., Sellwood E., Raftery J., Rösler M., Selai C.E., Trimble M.R., Rossor M.N., Harvey R.J., Storosum J.G. (1999). Effectiveness of rivastigmine in Alzheimer’s disease. BMJ.

[53] Fraser M.D., Davies J.R.T., Chang X. (2017). New Gold in Them Thar Hills: Testing a Novel Supply Route for Plant-Derived Galanthamine. J. Alzheimers. Dis..

[54] De Souza F.M.S., Busquet N., Blatner M., Maclean K.N., Restrepo D. (2011). Galantamine improves olfactory learning in the Ts65Dn mouse model of Down syndrome. Sci. Rep..

[55] Tariot P.N., Solomon P.R., Morris J.C., Kershaw P., Lilienfeld S., Ding C. (2000). A 5-month, randomized, placebo-controlled trial of galantamine in AD. The Galantamine USA-10 Study Group. Neurology.

[56] Cummings J.L., Cyrus P.A., Bieber F., Mas J., Orazem J., Gulanski B. (1998). Metrifonate treatment of the cognitive deficits of Alzheimer’s disease. Metrifonate Study Group. Neurology.

[57] Schneider L.S., Giacobini E. (1999). Metrifonate: A cholinesterase inhibitor for Alzheimer’s disease therapy. CNS Drug Rev..

[58] Lilja A.M., Luo Y., Yu Q.S., Röjdner J., Li Y., Marini A.M., Marutle A., Nordberg A., Greig N.H. (2013). Neurotrophic and Neuroprotective Actions of (-)- and (+)-Phenserine, Candidate Drugs for Alzheimer’s Disease. PLoS ONE.

[59] Klein J. (2007). Phenserine. Phenserine. Expert Opin. Investig. Drugs.

[60] Nordberg A., Kadir A., Andreasen N., Almkvist O., Wall A., Blennow K., Langstrom B., Zetterberg H. (2015). Correlations between Alzheimer’s Disease Cerebrospinal Fluid Biomarkers and Cerebral Glucose Metabolism after 12 Months of Phenserine Treatment. J. Alzheimer Dis..

[61] Kamal M.A., Greig N.H., Alhomida A.S., Al-Jafari A.A. (2000). Kinetics of human acetylcholinesterase inhibition by the novel experimental alzheimer therapeutic agent, tolserine. Biochem. Pharmacol..

[62] Wang D., Zhang Q., Hu X., Wang W., Zhu X., Yuan Z. (2018). Pharmacodynamics in Alzheimer’s disease model rats of a bifunctional peptide with the potential to accelerate the degradation and reduce the toxicity of amyloid β-Cu fibrils. Acta Biomater..

[63] Zhan Z.J., Bian H.L., Wang J.W., Shan W.G. (2010). Synthesis of physostigmine analogues and evaluation of their anticholinesterase activities. Bioorganic Med. Chem. Lett..

[64] Wang R., Yan H., Tang X.-C. (2006). Progress in studies of huperzine A, a natural cholinesterase inhibitor from Chinese herbal medicine 1. Acta Pharmacol. Sin..

[65] Braga de Andrade Teles R., Coimbra Diniz T., Coimbra Costa Pinto T., Gonçalves de Oliveira Júnior R., Gama Silva M., Martins de Lavor É., Wilton Cavalcante Fernandes A., Paula de Oliveira A., Pires Rodrigues de Almeida Ribeiro F., Alves Marcelino da Silva A. (2018). Flavonoids as Therapeutic Agents in Alzheimer’s and Parkinson’s Diseases: A Systematic Review of Preclinical Evidences. Oxidative Med. Cell. Longevity.

[66] De Paula A.A.N., Martins J.B.L., dos Santos M.L., Nascente L.D.C., Romeiro L.A.S., Areas T.F.M.A., Vieira K.S.T., Gambôa N.F., Castro N.G., Gargano R. (2009). New potential AChE inhibitor candidates. Eur. J. Med. Chem..

[67] Taiwo E.A. (2015). Cashew Nut Shell Oil—A Renewable and Reliable Petrochemical Feedstock. Advances in Petrochemicals.

[68] Lemes L.F.N., De Andrade Ramos G., De Oliveira A.S., Da Silva F.M.R., De Castro Couto G., Da Silva Boni M., Guimarães M.J.R., Souza I.N.O., Bartolini M., Andrisano V. (2016). Cardanol-derived AChE inhibitors: Towards the development of dual binding derivatives for Alzheimer’s disease. Eur. J. Med. Chem..

[69] Rizzo S., Bartolini M., Ceccarini L., Piazzi L., Gobbi S., Cavalli A., Recanatini M., Andrisano V., Rampa A. (2010). Targeting Alzheimer’s disease: Novel indanone hybrids bearing a pharmacophoric fragment of AP2238. Bioorganic Med. Chem..

[70] Piazzi L., Rampa A., Bisi A., Gobbi S., Belluti F., Cavalli A., Bartolini M., Andrisano V., Valenti P., Recanatini M. (2003). 3-(4-{[benzyl(methyl)amino]methyl}-phenyl)-6,7-dimethoxy-2H-2-chromenone (AP2238) inhibits both acetylcholinesterase and acetylcholinesterase-induced β-amyloid aggregation: A dual function lead for Alzheimer’s disease therapy. J. Med. Chem..

[71] Agatonovic-Kustrin S., Kettle C., Morton D.W. (2018). A molecular approach in drug development for Alzheimer’s disease. Biomed. Pharmacother..

[72] Camps P., Formosa X., Galdeano C., Muñoz-Torrero D., Ramírez L., Gómez E., Isambert N., Lavilla R., Badia A., Clos M.V. (2009). Pyrano[3,2-c]quinoline—6-chlorotacrine hybrids as a novel family of acetylcholinesterase-and β-amyloid-directed anti-Alzheimer compounds. J. Med. Chem..

[73] Girek M., Szymański P. (2019). Tacrine hybrids as multi-target-directed ligands in Alzheimer’s disease: Influence of chemical structures on biological activities. Chem. Pap..

[74] Pi R., Mao X., Chao X., Cheng Z., Liu M., Duan X., Ye M., Chen X., Mei Z., Liu P. (2012). Tacrine-6-ferulic acid, a novel multifunctional dimer, inhibits amyloid-β-mediated Alzheimer’s disease-associated pathogenesis in vitro and in vivo. PLoS ONE.

[75] Fernández-Bachiller M.I., Pérez C., González-Muñoz G.C., Conde S., Lόpez M.G., Villarroya M., García A.G., Rodríguez-Franco M.I. (2010). Novel Tacrine−8-Hydroxyquinoline Hybrids as Multifunctional Agents for the Treatment of Alzheimer’s Disease, with Neuroprotective, Cholinergic, Antioxidant, and Copper-Complexing Properties. J. Med. Chem..

[76] De La Torre P., Saavedra L.A., Caballero J., Quiroga J., Alzate-Morales J.H., Cabrera M.G., Trilleras J. (2012). A novel class of selective acetylcholinesterase inhibitors: Synthesis and evaluation of (E)-2-(benzo[d]thiazol-2-yl)-3-heteroarylacrylonitriles. Molecules.

[77] El-Malah A., Gedawy E.M., Kassab A.E., Salam R.M.A. (2014). Novel tacrine analogs as potential cholinesterase inhibitors in Alzheimer’s disease. Arch. Pharm..

[78] Korabecny J., Musilek K., Holas O., Binder J., Zemek F., Marek J., Pohanka M., Opletalova V., Dohnal V., Kuca K. (2010). Synthesis and in vitro evaluation of N-alkyl-7-methoxytacrine hydrochlorides as potential cholinesterase inhibitors in Alzheimer disease. Bioorganic Med. Chem. Lett..

[79] Weinreb O., Amit T., Bar-Am O., Youdim M.B.H. (2011). A Novel Anti-Alzheimer Disease Drug, Ladostigil. Neuroprotective, Multimodal Brain-Selective Monoamine Oxidase and Cholinesterase Inhibitor.

[80] Birks J.S., Birks J.S. (2006). Cholinesterase inhibitors for Alzheimer’s disease. Cochrane Database of Systematic Reviews.

[81] Dou K.-X., Tan M.-S., Tan C.-C., Cao X.-P., Hou X.-H., Guo Q.-H., Tan L., Mok V., Yu J.-T. (2018). Comparative safety and effectiveness of cholinesterase inhibitors and memantine for Alzheimer’s disease: A network meta-analysis of 41 randomized controlled trials. Alzheimer Res. Ther..

[82] Zenaro E., Piacentino G., Constantin G. (2017). The blood-brain barrier in Alzheimer’s disease. Neurobiol. Dis..

[83] Lipsman N., Meng Y., Bethune A.J., Huang Y., Lam B., Masellis M., Herrmann N., Heyn C., Aubert I., Boutet A. (2018). Blood–brain barrier opening in Alzheimer’s disease using MR-guided focused ultrasound. Nat. Commun..

[84] Cai Z., Qiao P.F., Wan C.Q., Cai M., Zhou N.K., Li Q. (2018). Role of Blood-Brain Barrier in Alzheimer’s Disease. J. Alzheimer Dis..

[85] Montagne A., Zhao Z., Zlokovic B.V. (2017). Alzheimer’s disease: A matter of blood-brain barrier dysfunction?. J. Exp. Med..

[86] Sweeney M.D., Sagare A.P., Zlokovic B.V. (2018). Blood-brain barrier breakdown in Alzheimer disease and other neurodegenerative disorders. Nat. Rev. Neurol..

[87] Marques F., Sousa J., Sousa N., Palha J. (2013). Blood–brain-barriers in aging and in Alzheimer’s disease. Mol. Neurodegener..

[88] Kalaria R.N. (2000). Blood Brain Barrier Dysfunction and Cerebrovascular Degeneration in Alzheimer’s Disease. Cerebral Amyloid Angiopathy in Alzheimer’s Disease and Related Disorders.

[89] Banks W.A. (2012). Drug delivery to the brain in Alzheimer’s disease: Consideration of the blood-brain barrier. Adv. Drug Deliv. Rev..

[90] Matsui K., Taniguchi S., Yoshimura T. (1999). Correlation of the intrinsic clearance of donepezil (Aricept^®^) between in vivo and in vitro studies in rat, dog and human. Xenobiotica.

[91] Geerts H., Guillaumat P.O., Grantham C., Bode W., Anciaux K., Sachak S. (2005). Brain levels and acetylcholinesterase inhibition with galantamine and donepezil in rats, mice, and rabbits. Brain Res..

[92] Chakraborty A., de Wit N.M., van der Flier W.M., de Vries H.E. (2017). The blood brain barrier in Alzheimer’s disease. Vascul. Pharmacol..

[93] Zdarova Karasova J., Sestak V., Korabecny J., Mezeiova E., Palicka V., Kuca K., Mzik M. (2018). 1-Benzyl-4-methylpiperidinyl moiety in donepezil: The priority ticket across the blood-brain-barrier in rats. J. Chromatogr. B Anal. Technol. Biomed. Life Sci..

[94] Nordberg A., Svensson A.L. (1998). Cholinesterase inhibitors in the treatment of Alzheimer’s disease. A comparison of tolerability and pharmacology. Drug Saf..

[95] Wagstaff A.J., McTavish D. (1994). Tacrine: A Review of its Pharmacodynamic and Pharmacokinetic Properties, and Therapeutic Efficacy in Alzheimer’s Disease. Drugs Aging.

[96] Mishra P., Kumar A., Panda G. (2019). Anti-cholinesterase hybrids as multi-target-directed ligands against Alzheimer’s disease (1998–2018). Bioorganic Med. Chem..

[97] Zueva I., Dias J., Lushchekina S., Semenov V., Mukhamedyarov M., Pashirova T., Babaev V., Nachon F., Petrova N., Nurullin L. (2019). New evidence for dual binding site inhibitors of acetylcholinesterase as improved drugs for treatment of Alzheimer’s disease. Neuropharmacology.

[98] Pepeu G. (2001). Overview and perspective on the therapy of Alzheimer’s disease from a preclinical viewpoint. Prog. Neuro Psychopharmacol. Biol. Psychiatry.

[99] Kurz A., Perneczky R. (2011). Novel insights for the treatment of Alzheimer’s disease. Prog. Neuro Psychopharmacol. Biol. Psychiatry.

[100] Pepeu G. (2004). Mild cognitive impairment: Animal models. Dialogues Clin. Neurosci..

[101] Zhu H., Yan H., Tang N., Li X., Pang P., Li H., Chen W., Guo Y., Shu S., Cai Y. (2017). Impairments of spatial memory in an Alzheimer’s disease model via degeneration of hippocampal cholinergic synapses. Nat. Commun..

[102] Zilony-Hanin N., Rosenberg M., Richman M., Yehuda R., Schori H., Motiei M., Rahimipour S., Groisman A., Segal E., Shefi O. (2019). Neuroprotective Effect of Nerve Growth Factor Loaded in Porous Silicon Nanostructures in an Alzheimer’s Disease Model and Potential Delivery to the Brain. Small.

[103] Zhen J., Qian Y., Fu J., Su R., An H., Wang W., Zheng Y., Wang X. (2017). Deep brain magnetic stimulation promotes neurogenesis and restores cholinergic activity in a transgenic mouse model of Alzheimer’s disease. Front. Neural Circuits.

[104] Xia E., Xu F., Hu C., Kumal J.P.P., Tang X., Mao D., Li Y., Wu D., Zhang R., Wu S. (2019). Young Blood Rescues the Cognition of Alzheimer’s Model Mice by Restoring the Hippocampal Cholinergic Circuit. Neuroscience.

[105] Wang Y., Guan X., Chen X., Cai Y., Ma Y., Ma J., Zhang Q., Dai L., Fan X., Bai Y. (2019). Choline Supplementation Ameliorates Behavioral Deficits and Alzheimer’s Disease-Like Pathology in Transgenic APP/PS1 Mice. Mol. Nutr. Food Res..

[106] Park T.Y., Kim S.H., Shin Y.C., Lee N.H., Lee R.K.C., Shim J.H., Glimcher L.H., Mook-Jung I., Cheong E., Kim W.K. (2013). Amelioration of neurodegenerative diseases by cell death-induced cytoplasmic delivery of humanin. J. Control. Release.

[107] Kim M.J., Park S.Y., Lee S.H., Kim Y., Kim Y.J., Jun W., Yoon H.G. (2019). Ameliorative Effects of Dendropanax morbifera on Cognitive Impairment Via Enhancing Cholinergic Functions and Brain-Derived Neurotrophic Factor Expression in β-Amyloid-Induced Mice. J. Med. Food.

[108] Karthivashan G., Park S.Y., Kweon M.H., Kim J., Haque M.E., Cho D.Y., Kim I.S., Cho E.A., Ganesan P., Choi D.K. (2018). Ameliorative potential of desalted *Salicornia europaea* L. extract in multifaceted Alzheimer’s-like scopolamine-induced amnesic mice model. Sci. Rep..

[109] Sato T., Ohi Y., Kato D., Mizuno M., Takase H., Kanamori T., Borlongan C.V., Haji A., Matsukawa N. (2017). Hippocampal Cholinergic Neurostimulating Peptide as a Possible Modulating Factor against Glutamatergic Neuronal Disability by Amyloid Oligomers. Cell Transplant..

[110] Ko Y.H., Kim S.Y., Lee S.Y., Jang C.G. (2018). 6,7,4′-Trihydroxyisoflavone, a major metabolite of daidzein, improves learning and memory via the cholinergic system and the p-CREB/BDNF signaling pathway in mice. Eur. J. Pharmacol..

[111] Reale M., D’Angelo C., Costantini E., Di Nicola M., Yarla N.S., Kamal M.A., Salvador N., Perry G. (2018). Expression Profiling of Cytokine, Cholinergic Markers, and Amyloid-β Deposition in the APP SWE /PS1dE9 Mouse Model of Alzheimer’s Disease Pathology. J. Alzheimer Dis..

[112] Karami A., Eriksdotter M., Kadir A., Almkvist O., Nordberg A., Darreh-Shori T. (2019). CSF Cholinergic Index, a New Biomeasure of Treatment Effect in Patients With Alzheimer’s Disease. Front. Mol. Neurosci..

[113] Hall H., Iulita M.F., Gubert P., Flores Aguilar L., Ducatenzeiler A., Fisher A., Cuello A.C. (2018). AF710B, an M1/sigma-1 receptor agonist with long-lasting disease-modifying properties in a transgenic rat model of Alzheimer’s disease. Alzheimer Dement..

[114] McKeever P.M., Kim T.H., Hesketh A.R., MacNair L., Miletic D., Favrin G., Oliver S.G., Zhang Z., St George-Hyslop P., Robertson J. (2017). Cholinergic neuron gene expression differences captured by translational profiling in a mouse model of Alzheimer’s disease. Neurobiol. Aging.

[115] Hollnagel J.O., Elzoheiry S., Gorgas K., Kins S., Beretta C.A., Kirsch J., Kuhse J., Kann O., Kiss E. (2019). Early alterations in hippocampal perisomatic GABAergic synapses and network oscillations in a mouse model of Alzheimer’s disease amyloidosis. PLoS ONE.

[116] Llorente-Ovejero A., Manuel I., Lombardero L., Giralt M.T., Ledent C., Giménez-Llort L., Rodríguez-Puertas R. (2018). Endocannabinoid and Muscarinic Signaling Crosstalk in the 3xTg-AD Mouse Model of Alzheimer’s Disease. J. Alzheimer Dis..

[117] Knez D., Coquelle N., Pišlar A., Žakelj S., Jukič M., Sova M., Mravljak J., Nachon F., Brazzolotto X., Kos J. (2018). Multi-target-directed ligands for treating Alzheimer’s disease: Butyrylcholinesterase inhibitors displaying antioxidant and neuroprotective activities. Eur. J. Med. Chem..

[118] Kumar A., Tiwari A., Sharma A. (2018). Changing Paradigm from one Target one Ligand Towards Multi-target Directed Ligand Design for Key Drug Targets of Alzheimer Disease: An Important Role of In Silico Methods in Multi-target Directed Ligands Design. Curr. Neuropharmacol..

[119] Bolognesi M., Minarini A., Rosini M., Tumiatti V., Melchiorre C. (2008). From Dual Binding Site Acetylcholinesterase Inhibitors to Multi-Target-Directed Ligands (MTDLs): A Step Forward in the Treatment of Alzheimers Disease. Mini Rev. Med. Chem..

[120] Mohamed T., Shakeri A., Rao P.P.N. (2016). Amyloid cascade in Alzheimer’s disease: Recent advances in medicinal chemistry. Eur. J. Med. Chem..

[121] Bajda M., Guzior N., Ignasik M., Malawska B. (2011). Multi-Target-Directed Ligands in Alzheimer’s Disease Treatment. Curr. Med. Chem..

[122] Oliveira C., Cagide F., Teixeira J., Amorim R., Sequeira L., Mesiti F., Silva T., Garrido J., Remião F., Vilar S. (2018). Hydroxybenzoic Acid Derivatives as Dual-Target Ligands: Mitochondriotropic Antioxidants and Cholinesterase Inhibitors. Front. Chem..

[123] Yu Q.S., Holloway H.W., Utsuki T., Brossi A., Greig N.H. (1999). Synthesis of novel phenserine-based-selective inhibitors of butyrylcholinesterase for Alzheimer’s disease. J. Med. Chem..

[124] Unzeta M., Esteban G., Bolea I., Fogel W.A., Ramsay R.R., Youdim M.B.H., Tipton K.F., Marco-Contelles J. (2016). Multi-target directed donepezil-like ligands for Alzheimer’s disease. Front. Neurosci..

[125] Lu C., Guo Y., Yan J., Luo Z., Luo H.B., Yan M., Huang L., Li X. (2013). Design, synthesis, and evaluation of multitarget-directed resveratrol derivatives for the treatment of Alzheimer’s disease. J. Med. Chem..

[126] Lee H.Y., Fan S.J., Huang F.I., Chao H.Y., Hsu K.C., Lin T.E., Yeh T.K., Lai M.J., Li Y.H., Huang H.L. (2018). 5-Aroylindoles Act as Selective Histone Deacetylase 6 Inhibitors Ameliorating Alzheimer’s Disease Phenotypes. J. Med. Chem..

[127] Vohora D., Bhowmik M. (2012). Histamine H3 receptor antagonists/inverse agonists on cognitive and motor processes: Relevance to Alzheimer’s disease, ADHD, schizophrenia, and drug abuse. Front. Syst. Neurosci..

[128] Gemkow M.J., Davenport A.J., Harich S., Ellenbroek B.A., Cesura A., Hallett D. (2009). The histamine H3 receptor as a therapeutic drug target for CNS disorders. Drug Discov. Today.

[129] Berlin M., Boyce C.W., De Lera Ruiz M. (2011). Histamine H3 receptor as a drug discovery target. J. Med. Chem..

[130] Muñoz-Ruiz P., Rubio L., García-Palomero E., Dorronsoro I., Del Monte-Millán M., Valenzuela R., Usán P., De Austria C., Bartolini M., Andrisano V. (2005). Design, synthesis, and biological evaluation of dual binding site acetylcholinesterase inhibitors: New disease-modifying agents for Alzheimer’s disease. J. Med. Chem..

[131] Lange J.H.M., Coolen H.K.A.C., Van Der Neut M.A.W., Borst A.J.M., Stork B., Verveer P.C., Kruse C.G. (2010). Design, synthesis, biological properties, and molecular modeling investigations of novel tacrine derivatives with a combination of acetylcholinesterase inhibition and cannabinoid CB1 receptor antagonism. J. Med. Chem..

[132] Smith T.H., Sim-Selley L.J., Selley D.E. (2010). Cannabinoid CB 1 receptor-interacting proteins: Novel targets for central nervous system drug discovery?. Br. J. Pharmacol..

[133] Ghosh A.K., Osswald H.L. (2014). BACE1 (β-secretase) inhibitors for the treatment of Alzheimer’s disease. Chem. Soc. Rev..

[134] Prati F., Bottegoni G., Bolognesi M.L., Cavalli A. (2018). BACE-1 Inhibitors: From Recent Single-Target Molecules to Multitarget Compounds for Alzheimer’s Disease. J. Med. Chem..

[135] Wu H., Li X.D., Peng D.T., Jiao J.S., Li Y.F., Yu P.L., Ji Y. (2017). The role of butyrylcholinesterase in the pathogenesis of Alzheimer’s disease. Chin. J. Contemp. Neurol. Neurosurg..

[136] Wu Y., Li Z., Huang Y.Y., Wu D., Luo H. (2018). Bin Novel Phosphodiesterase Inhibitors for Cognitive Improvement in Alzheimer’s Disease. J. Med. Chem..

[137] Vila-Real H., Coelho H., Rocha J., Fernandes A., Ventura M.R., Maycock C.D., Iranzo O., Simplício A.L. (2015). Peptidomimetic β-Secretase Inhibitors Comprising a Sequence of Amyloid-β Peptide for Alzheimer’s Disease. J. Med. Chem..

[138] Benek O., Hroch L., Aitken L., Gunn-Moore F., Vinklarova L., Kuca K., Perez D.I., Perez C., Martinez A., Fisar Z. (2018). 1-(Benzo[d]thiazol-2-yl)-3-phenylureas as dual inhibitors of casein kinase 1 and ABAD enzymes for treatment of neurodegenerative disorders. J. Enzyme Inhib. Med. Chem..

[139] Aitken L., Benek O., McKelvie B.E., Hughes R.E., Hroch L., Schmidt M., Major L.L., Vinklarova L., Kuca K., Smith T.K. (2019). Novel Benzothiazole-based Ureas as 17β-HSD10 Inhibitors, A Potential Alzheimer’s Disease Treatment. Molecules.

